# The impact of body mass index on short-term surgical outcomes after laparoscopic hepatectomy, a retrospective study

**DOI:** 10.1186/s12871-016-0194-1

**Published:** 2016-06-04

**Authors:** Xin Yu, Hong Yu, Xiangming Fang

**Affiliations:** 1 Department of Anaesthesiology, Sir Run Run Shaw Hospital, School of Medicine, Zhejiang University, Hangzhou, China; 2 Department of General Surgery, Sir Run Run Shaw Hospital, School of Medicine, Zhejiang University, Hangzhou, China; 3 Department of Anesthesiology, the First Affiliated Hospital, School of Medicine, Zhejiang University, Qing Chun Road 79, Hangzhou, 310003 Zhejiang China

**Keywords:** Body mass index, Laparoscopic hepatectomy, Short-outcomes, Conversion rate, Complications

## Abstract

**Background:**

Surgeons may expect technical difficulties and worse outcomes when performing laparoscopic hepatectomy (LH) on obese patients. The aim of this study is to assess the impact of body mass index (BMI) on short-term surgical outcomes and to verify risk factors of conversion rate and complications of LH.

**Methods:**

Data were collected from 551 patients who underwent attempted LH between August 1998 and April 2013. Patients were classified into four groups depending on their BMI according to the WHO’s definition of obesity for Asia-Pacific region: underweight <18.5 kg/m^2^ (Group1); normal 18.5–23.9 kg/m^2^ (Group2); overweight 24–27.9 kg/m^2^ (Group3); obese ≥ 28 kg/m^2^ (Group4) respectively. Short-term surgical outcomes were compared across the BMI categories. Possible risk factors concerned conversion rate and complications were analyzed.

**Results:**

The overall conversion rate of the 551 patients was 13.07 %. Conversion rate for Group 1, 2, 3, and 4 were 14.3 % (*n* = 5), 11.2 % (*n* = 38), 13.0 % (*n* = 19), and 34.5 % (*n* = 10) respectively. Patients within the obese group had a much higher conversion rate. The overall complications rate was 11.98 %, where the complication for Group 1, 2, 3, and 4 were 22.9 % (*n* = 8), 12.7 % (*n* = 43), 18.2 % (*n* = 12), and 10.3 % (*n* = 3) respectively. Patients within the underweight group had a higher complication rate, but it did not reach statistic difference. Obesity and surgical site of left lobe were independent risk factors of conversion. Age, abdominal surgery history, and type of left and right lobe resection were independent risk factors for complications.

**Conclusions:**

In China, obesity increases risk of conversion rate but it dose not affect surgical complications and other short-outcomes after LH.

**Electronic supplementary material:**

The online version of this article (doi:10.1186/s12871-016-0194-1) contains supplementary material, which is available to authorized users.

## Background

Since the first laparoscoic liver resection was reported in 1992 [1], it is used from limited resection toward major hepatectomy. A number of advantages have been established when compared with conventional liver surgery, including less postoperative pain, shorter length of postoperativer hospital stay, and faster recovery of daily activities [2–4]. However due to the special anatomic position and massive vascularity of liver parenchyma, LH is still as one of the last barriers to laparoscopic surgery. Risk of massive bleeding, high rate of conversion and complications are difficulties to be solved.

Obesity, a growing public health problem worldwide, although is less common in China, it has a rapidly growing trend in recent decades. The total prevalence rate of overweight and obesity was up to 42.6 % of Chinese adults [4–6]. Overweight and obesity have been reported being associated with metabolic syndrome, diabetes, hypertension, and increased risk of surgical infection [7]. Surgeons may expect difficulties such as inadequate exposure and technical challenges in performing LH on overweight and obese patients. By now there are no so many studies focusing on risk factors of conversion and complications and little information is currently available around the influence of obesity on outcomes of LH. In the present study we firstly try to compare the short-term outcomes after LH in relation to BMI that is now widely recognized as a reliable indicator to describe the extent of obesity and is easily calculated.

## Methods

### Patients and data collection

In this retrospective study, 554 consecutive patients who underwent LH between 1998 and 2013 at Sir Run Run Shaw Hospital were identified from patients database, three patients died during the postoperative period were excluded.

According to the WHO’s definition of obesity for the Asia-Pacific region [8], 551 Patients were segregated into four groups by BMI: underweight <18.5 kg/m^2^ (Group1); normal weight 18.5–23.9 kg/m^2^ (Group 2); overweight 24–27.9 kg/m^2^ (Group 3); obese ≥28 kg/m^2^ (Group 4).

BMI was calculated according to a standardized definition as weight in kilograms divided by height in meters squared. BMI was recorded the day before the surgery.

Data collection included standard demographic information (age, gender, height, and weight), American Society of Anesthesiologists (ASA) score, prior abdominal surgery, diabetes, conversion, operation duration, blood loss, transfusion (Hemoglobin < 7 g/dl as the indication), type of disease, type of resection, pathology, complications (those that occurred at any time during the postoperative hospital stay), postoperative length of stay (LOS), and severity of complication which were categorized according to the Clavien-Dindo classification system [9]. In the analysis, grades 1 and 2 were considered as minor complications, whereas grades 3–5 were considered as major complications. And only the highest ranked complication was chosen for the final analysis.

Liver resection cases were categorized according to Couinaud's classification as follows [10]: (1) Left hepatectomy for resection of segments II–IV; (2) Right hemihepatectomy for resection of segments V–VIII; (3) Left lateral hepatectomy for resection of segments II and III; (4) Segmentectomy for resection of a single segment; (5) Local hepatectomy for resection of less than a single segment. (6) Caudate for resection of segment I.

### Surgical procedure

The indication of laparoscopic treatment for liver disease was made during a multidisciplinary conference, which included hepatobiliary surgeons, radiologists, as well as patient’s preference.

The procedure was performed with the patient in the supine and 30° anti-Trendelenburg position under carbon dioxide pneumoperitoneum, the abdominal pressure was maintained between 12 and 15 mmHg. For a right hemihepatectomy, the patient would be positioned in the 45°right side cushion with the table turned to its left side.

A 10-mm trocar was positioned above the umbilicus for insertion of optical device and the linear stapler. A 12-mm port was positioned on the crossing of left midcalvicular line and costal margin for surgical aspirator or harmonic scissors. Other two 5-mm ports were inserted in the left upper abdominal quadrant according to the lesion location, allowing the assistant aspirate, irrigate or hang the live for a better exposure.

Instead of total hepatic vascular occlusion, regional occlusion of liver left/right inflow and outflow was used to reduce bleeding and minimize the ischemia reperfusion injury [11]. The pringle maneuver was used only when there was bleeding.

Liver parenchymal transection and small vascular disconnection were almost performed with multifunctional electric knife LPMOD (laparoscopic multiple operation dissector) which can scrape the hepatic parenchyma, separate and dissect vessels and bile ducts of liver section, and allow suction of blood and smoke to provide a clear view [12]. The operation was considered to convert if there was an unsatisfactory visualization, an uncontrolled bleeding, difficult manipulation, or unclear tumor edge.

### Statistical analysis

Data are presented as the mean ± SD for normal distribution, and the median with an interquartile range for non-normally distributed parameters. Continuous data normally distributed were compared using the two-side Student’s *t-test*. Continuous data non-normally distributed were compared using the Kruskal-Wallis test. Comparisons between groups for categorical variables were performed using the ANOVA analysis with Fisher’s exact test, as appropriate. To identify variables that were independent predictors of conversion rate and complications, only factors associated with conversion rate and complications in the univariate analysis with significant difference entered into a logistic regression analysis. 95 % confident interval (CI) and odd ratios (OR) were calculated. Significance was defined as *p* < 0.05. All statistic analyses were made with SPSS Version18.

## Results

### Patients

In the whole cohort, three patients died during the postoperative period. The mortality was 0.54 %. 551 patients over 15 years were identified, there were 37 (6.72 %) underweight (<18.5 kg/m^2^), 339 (61.52 %) normalweight (18.5–23.9 kg/m^2^), 146 (26.50 %) overweight (24–27.9 kg/m^2^), 29 (5.26 %) obese (≥28 kg/m^2^). 175(31.76 %) patients were overweight and obese. Patient baseline demographics and pathological variables were compared between the groups (Table 1). There was no statistically significant difference across the 4 BMI groups with respect to age, gender, ASA score, diabetes incidence, and abdominal operation history.

**Table 1:** Demographics, pathological and surgical variable in BMI groups of 551 patients under LH (operation details in the four groups)

	Total	Group1	Group2	Group3	Group4 ≥ 28 kg/m^2^	*P* value
		<18.5 kg/m^2^	18.5-23.9 kg/m^2^	24-27.9 kg/m^2^		
	*N* = 551	*N* = 37	*N* = 339	*N* = 146	*N* = 29	
Age (years)	51.00 ± 12.69	47.46 ± 14.60	51.06 ± 12.82	51.71 ± 12.40	51.24 ± 9.65	0.340
Female/Male	311/240	26/11	186/153	83/63	16/13	0.355
ASAI	144	10	83	43	8	0.933
ASAII	377	25	237	96	19	0.601
ASAIII	29	2	19	6	2	0.637
ASAIV	1	0	0	1	0	0.905
Diabetes	20	1	13	5	1	0.985
Operative time(min, mean ± SD)						
	165.83 ± 93.63	179.97 ± 134.68	168.58 ± 91.41	159.22 ± 82.30	148.97 ± 109.90	0.424
Postoperative length of stay (min, mean ± SD)						
	9.82 ± 6.25	8.24 ± 3.56	9.81 ± 6.07	9.76 ± 5.83	12.34 ± 10.92	0.068
Postoperative length of stay of conversion patients (min, mean ± SD)						
	13.90 ± 7.6	8.20 ± 2.17	14.29 ± 7.20	12.68 ± 3.40	18.00 ± 13.91	0.002^*^
Estimated blood loss (ml, mean ± SD)						
	468.26 ± 62	448.65 ± 595.34	480.81 ± 621.91	424.01 ± 560.17	569.31 ± 920.84	0.179
Abdominal operation history						
	174(31.58 %)	13(37.14 %)	103(30.38 %)	49(33.56 %)	9(31.03 %)	0.457
Blood transfusion	88(15.97 %)	3(8.1 %)	50(14.7 %)	29 (19.9 %)	6(20.7 %)	0.240
Complication	66(11.98 %)	8 (22.86 %)	43(12.68 %)	12 (8.22 %)	3(10.34 %)	0.142
Severity of complication (Clavien -Dindo)						
I–II/III–IV	51/15	8/0	31/12	10/2	2/1	0.354
Conversion	72/551(13.1 %)	5(13.50 %)	39(11.5 %)	19(13.0 %)	9(31.0 %)	0.030*
Type of surgery						
Wedge resection	119	7	68	33	11	0.388
Segmenttectomy	74	2	50	19	3	0.000^*^
Left lateral hepatectoy	184	17	108	48	11	0.229
Left hemihepatectomy	144	10	92	39	3	0.000^*^
Right hemihepatectoy	23	1	16	5	1	0.221
Caudate hepatectomy	7	0	5	2	0	0.088
Diagnosis						
Maliganmacy/Benign	141/410	10/27	88/251	35/111	8/21	0.955
Maliganmacy						
Hepatocellular carcinoma	99	5	64	25	5	0.613
Metastatic hepatic	23	2	13	6	2	0.941
Carcinoma	11	1	8	1	1	0.574
Chalangiocarcinoma	9	0	7	1	1	0.483
Benign						
Hepatic hemangioma	162	10	95	45	12	0.341
Intrahepatic stone	191	15	117	53	6	0.503
Focalnodularhyperplasa	27	2	19	5	1	0.756
Hepatic cyst	13	1	6	6	0	0.376
Adenoma	8	1	4	2	1	0.702
Others	8	0	6	2	0	0.750

Values are presented as mean ± SD, median (percentage)

*ASA* American Society of Anesthesiology

*P<0.05

### Short-term perioperative outcomes

The short perioperative outcomes were also shown in Table 1. The overall conversion rate was 13.1 %. Conversion rates for groups 1, 2, 3, and 4 were 13.5 % (*n* = 5), 11.5 % (*n* = 39), 13.0 % (*n* = 19), and 31.0 % (*n* = 9) respectively. Patients in the obese group were associated with a much higher conversion rate than the other three groups (*p* = 0.03). The total combined complications rate is 12.0 % in the present study, Complication for groups 1, 2, 3, and 4 were 22.86 % (*n* = 8), 12.68 % (*n* = 43), 8.22 % (*n* = 12), and 10.34 % (*n* = 3) respectively. Although there was a higher rate of complication in underweight group, overall complication rates did not differ significantly among the groups. No significant differences were observed in terms of operation duration, blood loss, blood transfusion, and postoperative LOS in the four groups. Obese patients had a longer postoperative LOS once converted to open approach (*p* = 0.002).

The complication rates according to the Clavien classification were reported in Table 1; they were 9.26 % for grade I to II and 2.72 % for grade III to IV. There was no grade V complication noted in any of the patients in this study. And no significant differences were observed in terms of the Clavien-Dindo classification among the four groups.

Reasons for conversion are shown in the Table 2. The top three reasons to conversion were unclear exposure, adhesions, and bleeding, by order of significance. There was a rare complication of diaphragm perforation that occurred, and the incidence rate was 1.39 %.

**Table 2:** Reasons for conversion

Reasons for conversion	Number	Value (% of conversion)
Total	72	100.00 %
Bleeding	19	26.39 %
Unclear expose	30	41.67 %
Adhesions	20	27.78 %
Unclear tumor margin	2	2.78 %
Other (diaphragm puncture)	1	1.39 %

Values are presented as percentage

Surgical time and blood loss of different type of resection are listed in Table 3. Patients underwent right hemihepatectomy had the longest operative time and with the largest amount of bleeding. And longer time was needed in patients underwent left hemihepatectomy.

**Table 3:** Surgical Time and Blood loss of different type of resection

Type of resection	Surgical time		Blood loss
	*N*	(minutes)	(ml)
Wedge resection	119	105.00	150.00
Segmentectomy	74	130.00	400.00
Left lateral hepatectomy	184	145.00	250.00
Left hemihepatectomy	144	192.50	300.00
Right hemihepatectomy	23	265.00	800.00
Caudate hepatectomy	7	180.00	280.00
		*P* = 0.000*	*P* = 0.000*

Values are presented as median *Kruskal-Wallis Test

The types of complications were listed in the Table 4. Only the incidence of  bile leakage was much higher in underweight group than that in other groups. There was no difference in the distribution of other complications in the four groups.

**Table 4:** Type of complications

Complications	Group1 <18.5 kg/^2^	Group2 18.5-23.9 kg/m^2^	Group3 24–27.9 kg/m^2^	Group4 ≥28 kg/m^2^	*P* value
*N* = 551	*N* = 37	*N* = 339	*N* = 146	*N* = 29	ns
Reoperation	0 (0.00)	0 (0.00)	2(1.37)	0(0.00)	ns
Blood	1 (2.86)	4 (1.18)	3(2.05)	0(0.00)	ns
Wound infection	1 (0.00)	4 (1.18)	1(0.68)	0(0.00)	ns
Bile leak	2(5.71)	4 (1.18)	0(0.00)	1(3.44)	<0.05
Intra-abdominal infection	1(5.71)	12(3.53)	5(3.42)	1(1.69)	ns
Ascites	1 (2.86)	4(1.18)	0(0.00)	0(0.00)	ns
Pneumonia	0(0.00)	5(1.47)	1(0.68)	0(0.00)	ns
Pleural effusion	0(0.00)	4 (1.18)	0(0.00)	0(0.00)	ns
Pulmonary embolus	0(0.00)	2(0.59)	1(0.68)	0(0.00)	ns
Angiocholitis	0(0.00)	0(0.00)	1(0.68)	0(0.00)	ns
Hepatic encephalopathy	0(0.00)	1(0.30)	0(0.00)	0(0.00)	ns
Urinary tract infection	0(0.00)	1(0.30)	0(0.00)	0(0.00)	ns
Hypoproteinemia	1 (2.86)	2(0.59)	0(0.00)	0(0.00)	ns
Trial fibrillation	1(2.86)	1(0.30)	0(0.00)	0(0.00)	ns
Septic shock	1(2.86)	0(0.00)	1(0.68)	1(1.69)	ns

Values are presented as median (range)/(percentage)

Independent factors for conversion and total complications were reported in Table 5. According to logistic analysis,conversion was strongly correlation to obesity and left hepatcetomy. Obesity and left hepatectomy were identified as a predictive factor of conversion [odd ratio (OR) = 5.06, 95 % confidence interval (CI) = 2.02–12.76, *P* = 0.001; OR = 3.4, 95 % CI = 1.36 ~ 11.45, *P* = 0.012 respectively]. Age, abdominal operation history, and left and right hepatectomy were identified as independent predictive factor for complications (OR = 1.03, 95 % CI = 1.01 ~ 1.54, *P* = 0.013; OR = 1.03, 95 % CI = 1.01 ~ 1.54, *P* = 0.013; OR = 4.39, 95 % CI = 1.62 ~ 11.89, *P* = 0.004; OR = 5.84, 95 % CI = 1.43 ~ 23.85, *P* = 0.014 respectively).

**Table 5:** Independent factors for conversion and total complications according to logistic analysis

Factor	Conversion			Complications		
	OR	95 % CI	*P*	OR	95 % CI	*P*
Age	1.29	0.69 ~ 2.40	0.560	1.03	1.01 ~ 1.54	0.013^*^
BMI						
< 18.5	1.34	0.48 ~ 3.90	0.560	2.00	0.82 ~ 4.86	0.128
18.5 ~ 23.9	1			1		
24 ~ 27.9	1.24	0.68 ~ 2.28	0.481	0.61	0.31 ~ 1.21	0.158
≥ 28	5.06	2.02 ~ 12.76	0.001^*^	0.99	0.27 ~ 3.68	0.990
Left lobe	3.40	1.36 ~ 11.45	0.012^*^	4.39	1.62 ~ 11.89	0.004^*^
Right lobe	1.13	0.50 ~ 2.58	0.770	5.84	1.43 ~ 23.85	0.014^*^
History of surgery	0.91	0.51 ~ 1.65	0.762	1.81	1.03 ~ 3.20	0.040^*^

*P<0.05

## Discussion

In the present study 31.76 % (175/551) of patients were overweight and obese. We identify obesity as the most important risk factor for conversion mainly for inadequate exposure of the surgical field and more surgical blood loss and a longer operation time were needed in the obesity patients. However our findings were not consistent with Troisi et al. analysis [13]. Maybe comparing with non-Asians, Asian population with the same BMI is considered to have a greater volume of intraperitoneal fat than subcutaneous fat and with more difficulty to expose the upper part of the transection plane [14]. Although, conversion could not been considered as a failure of the laparoscopic approach, this makes some of the benefits lost. In this setting, conversion was found associated with a longer hospital stay compared with fully laparoscopic liver resection. Interestingly, there was no difference in complications between patients who underwent conversion and those who did not. It was likely that we use Clavien-Dindo classification system, which could not clearly reflect conversion related wound and pulmonary infection. And there would be a bias due to retrospective analysis. The conversion rate in present study was 13.1 % (*n* = 72), which corresponds to rates described in the literature [13], [15]. The main reason for conversion was unclear exposure (41.67 %, 5.4 % of all patients), and the second cause was adhesion (26.39 %, 3.4 % of all patients). Our findings are not consistent with the most opinions that bleeding is the main technical difficulty [13, 16, 17]. This may be attributed to our special laparoscopic skill of regional occlusion of liver inflow and outflow, and well-scraping and coagulating function of LPMOD, to providing a clear view [10, 18]. Another explanation could be that there was little portion of major liver resection enrolled in this study that was much more difficult to control bleeding laparoscopically.

Not inline with former studies that obese patients have a worse outcome than their leaner counterparts [19, 20], our results showed that overweight and obese patients had a lower complication rate (8.57 %) than the overall complication rate (11.98 %). One causes may be that obesity has a protective effect for adequate fat storage, better nutrition,and systemic insulin resistance that underweight people do not have [21, 22]. Another possible causes may be the BMI could not adequately reflect adiposity for Asian people, who had more body fat than Europeans [13]. However, the patients in the underweight group had an increased rate of complications (22.86 %), but the increase did not demonstrate significance. Patients with a low BMI may be with nutritional damage, possible immune deficiency and a lower physiologic reserve and thus can’t withstand the hepatic resection coupled with other therapies. Also weight loss or low serum albumin levels of liver disease would be contributed to the complications.

Our study also aimed to verify the risk factors of conversion and complications. And our data revealed that obesity and surgical site of left lobe were the risk factors of conversion; abdominal surgery history and type of left and right hepatectomy were the risk factors of complication. The major finding was that conversion rate was statistically significant associated with left hemihepatectomy which also had a bit longer surgical time. For patients that we enrolled over the 15 years, the selected criteria were mostly left lateral lobe and left lobe at the early stage. Undoubtedly, a significant learning curve would account for initially higher conversion rates. And the left liver lobe is the most frequent location of hepatolithiasis, for there is an acute angle when the left hepatic duck reach the common hepatic duct [23, 24]. Thus, it will take more time for surgeons to deal with the stones. But the right lobectomy, which is considered the most technically challenging with uncontrollable bleeding, did not increase conversion rate. One possible explanation is that for the small number of right lobectomy patients enrolled, surgeons attempted to choose laparoscopic approach to right hemihepatectomy until they have experienced a learning curve. In our study we found right and left hemihepatectomy were the independent predictive factors of complications. It is easy to understand laparoscopic approach to right hemihepatectomy with higher complication rate is due to the less hepatic reserve and uncontrolled bleeding. Interestingly, our result showed a significantly greater proportion of complications occurred in the laparoscopic left hemihepatectomy. This observation may be accounted for the fact that the greater number of left lobectomy patients were with hepatilithiasis, which is easy combined with biliary tract infection, leading to bile leakage and infection postoperatively.

Not compatible with the conventional view that prior abdominal operation would increase the incidence of conversion rate duo to the sequel of peritoneal adhesions, in the present study we discovered that abdominal surgery history did not increase conversion rate but increaseed complications. Maybe more proportion of patients with prior abdominal surgery history occurred in the right hepatectomy was the possible cause of higher complication rate.

Moreover, we found that postoperative LOS was significantly superior in converted patients of obese group. We speculate that although obesity did not increase the total complications after LH, it may delay the healing process of the open-wound and increase the postoperative hospitalization for its potential increased tension on the abdominal incision and tissue hypoperfusion [6]. As highlighted by this finding, laparoscopy approach compared with open liver resection in obese patients can speed up prognosis and shorten hospitalization.

Our limitation is that the data have been retrospectively analyzed and they were from a single institution. The results of present study pertain to patients from China and hence may not be generalized to other populations. It was a retrospective observational and with selection bias, which may cloud the significant differences in outcome. We only used BMI to estimate obesity, which was not enough to assess the abdominal adiposity of Asian people.

We would also like to emphasize that our findings maybe not applicable to all the surgeons, for there were only three surgeons in a single team that performed all procedures.

## Conclusion

Our data showed that obesity would increase the conversion of LH, but it does not affect surgical safety. Obesity should not deter a surgeon from selecting a laparoscopic approach for liver resection surgery, but is still technically challenging and should be performed by experienced expert surgeons.

## Abbreviations

ASA, American Society of Anesthesiologists; BMI, Body mass index; CI, Confident interval; LH, Laparoscopic hepatectomy; LOS, Length of stay; LPMOD, Laparoscopic multiple operation dissector; OR, Odd ratios.

## Additional file

Additional file 1: Available data. (XLSX 107 kb)
